# Prone Position Airway Management of a Child following Penetrating Trauma to the Back

**DOI:** 10.1155/2022/3753415

**Published:** 2022-12-13

**Authors:** Mae Richelle S. Magbitang, Corinna J. Ongaigui

**Affiliations:** Department of Anesthesiology, Bicol Medical Center, Naga, Camarines Sur, Philippines

## Abstract

Traumatic penetrating injuries to the back are uncommon in children. This type of injury presents many considerations for airway management to the anesthesiologist, including having to secure the airway in a prone position. Although there have been several reports about intubation in the prone position for adult patients in emergency conditions, such studies on pediatric patients are rare. We present the case of a male child with an impaled steel shaft connected to a toy car wheel in his lower back, requiring an emergent operation under general anesthesia. Due to resource limitations, the patient was intubated using an adult-sized video laryngoscope in the prone position. The patient remained stable during the operation and was discharged without complications. A postoperative discussion was held later to review the case and gain insights from the rest of the anesthesiology team. Prone intubation in pediatric patients can be safely accomplished using various techniques, depending on the urgency of the need, the availability of resources, and the knowledge and skills of the provider. The authors hope that their colleagues can learn from sharing this experience.

## 1. Background

Trauma patients presenting with penetrating back injuries may require anesthesia and airway management in the prone position to avoid further complications or possible neurological impairment. Securing the airway of a child in the prone position and in an emergency situation requires many factors for anesthesiologists to consider, especially in a resource-limited setting. We herein present our experience, which brought up some specific anesthesia-related challenges not found in the literature, specifically in a child with a penetrating back injury requiring emergency surgery while in the prone position.

## 2. Case Presentation

**Figure 1:**
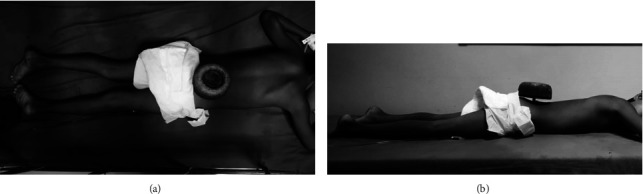
Above view (a) and side view (b) of the patient lying face down with a toy car wheel on an axle impaled on the lower lumbar region.

**Figure 2:**
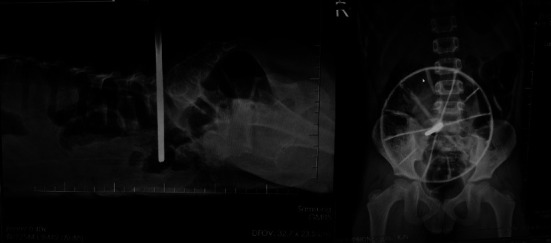
Radiographic imaging of the patient shows a radiopaque metallic rod and wheel seen at the right hemiabdomen immediately lateral to the L5 vertebrae with no apparent bone involvement at the time of the exam.

**Figure 3:**
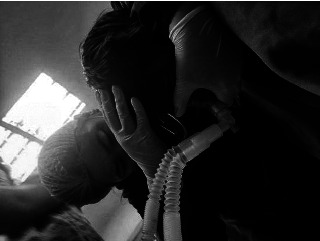
Preoxygenation is followed by a modified rapid-sequence induction using a one-handed technique.

**Figure 4:**
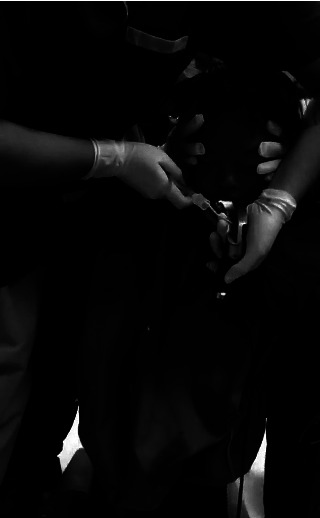
Laryngoscopy is performed from the side of the patient.

**Figure 5:**
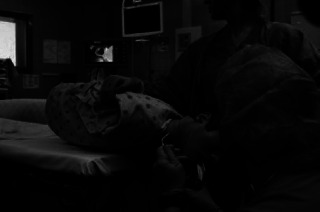
Image depicted is a near representation of the technique used on the second attempt to intubate the patient [17].

A 7-year-old male was transferred urgently to our emergency department with a penetrating injury to the back (Figure 1). He had been impaled by a steel shaft axle connected to a toy wheel, which pierced his lower back just lateral to his lumbosacral spine and entered his abdominal cavity (Figure 2). The unavailability of a CT scan facility in our hospital warranted prompt surgical exploration to accurately assess the injury's extent. The patient was lying prone and immobilized when brought to the operating room. He is conscious, breathing spontaneously, and hemodynamically stable. His neurologic examination was unremarkable. As the surgeons did not want to move or reposition the patient to avoid further injury, the anesthesiologists decided to secure the patient's airway in the prone position. The plan to intubate prone was discussed with the parents, including the steps that will be done and the alternative approaches to securing the airway should the first option fail. It was determined that the patient had had nothing per os for more than 8 hours. There were no features to suggest difficult mask ventilation or intubation. With the help of two assistants, the child's body was moved close to the head of the stretcher, suspending the patient's head and shoulders while keeping the head in a neutral position. After applying standard monitors, the patient was preoxygenated using a one-handed technique for three minutes (Figure 3). A modified rapid-sequence induction was carried out wherein, after IV administration of fentanyl at 1 mcg/kg and propofol at 2 mg/kg, succinylcholine at 2 mg/kg was administered only after verifying that the patient could be ventilated by gentle positive-pressure ventilation via a face mask. On the first attempt, video laryngoscopy using a C-MAC® (Karl Storz, Tuttlingen, Germany) video laryngoscope with a Macintosh size 4 blade was performed from the right side of the patient (Figure 4). The only other available blades were a Miller 1 and a D-Blade, and given that the Macintosh size 4 blade was too big for the child, the laryngoscope blade was only partially introduced. Because of a restricted view of the glottis, a second attempt was made after mask ventilation, this time with the child's head slightly tilted to the right by an assistant and the primary anesthesiologist facing the patient (Figure 5). The anesthesiologist (who squatted on the floor) performed intubation with his right hand holding the video laryngoscope and his left hand advancing the 5.5 mm ID PVC endotracheal tube (ETT) with a stylet through the vocal cords, as visualized on the C-MAC® monitor. The ETT was secured after confirmation of the ETT position and appropriate depth by auscultation and capnography. The patient was maintained on IV atracurium 0.5 mg/kg for muscle relaxation, and maintenance of anesthesia was done with sevoflurane with oxygen.IV fentanyl 1 mcg/kg was given for analgesia. The toy wheel with its axle was removed intact by the surgeon. The patient was then repositioned supine, and a careful abdominal exploratory laparotomy showed no internal organs were injured. No intraoperative problems were encountered. At surgery completion, the patient was extubated fully awake. His postoperative course was uneventful, and he was discharged from the hospital three days later.

## 3. Discussion

Tracheal intubation in the prone position following penetrating injuries to the back has been described, but only in adult patients [1–4]. Hence, we would like to share our experience managing a child's airway management in a similar clinical scenario and discuss considerations and options from a provider perspective in a resource-limited setting.

When the patient was initially referred to us, we thought about several considerations for our anesthetic approach. The first was that the trauma surgeons prohibited repositioning the patient, so attempts to secure the airway should be done in the prone position. Even if repositioning were allowed, the shape and size of the impaled object, the location of the impaled object (near the lumbosacral lordotic curve), and the required cooperation and compliance from the child might make repositioning difficult. Second, we considered the options available to us; flexible fiberoptic intubation (Karl Storz Flexible Intubation Video Endoscope, Series 11302 BDX) was excluded, given that nobody from the department is proficient in pediatric fiberoptic intubation, more so in an acute setting where securing the airway should be swift. The timing of airway intervention depends on the expertise of the provider. Performing a flexible fiberoptic intubation requires considerable training and experience to attain competency [5]. Additionally, experience with the flexible fiberoptic endoscope in adults does not equate to proficiency with flexible fiberoptic endoscopes in children [6, 7]. We also considered the insertion of a supraglottic airway (SAD) device. Still, we felt that the safer option was to undertake tracheal intubation via video laryngoscopy, which we were more adept at and could provide a more secured airway. The SAD was set aside as an alternative rescue plan in case of a failed intubation. The only available C-MAC® video laryngoscope (Karl Storz, Tuttlingen, Germany) blades in our institution are a Miller size 1 blade, D-Blade, and a Mac size 4 blade. A Mac size 2 blade is deemed appropriate for this patient, but because it was unavailable, we adapted the Mac 4 blade, carefully inserting only the thin, distal part, leaving plenty of room in the mouth to pass the endotracheal tube successfully.

The case was later presented to the department, which led to some debate and discussions on how to go about the airway management of the child. Given the limitations of our resources and expertise, most of our practitioners agreed that the most plausible approach was to utilize a SAD as a bridge for maintaining the airway until the patient is repositioned supine, where tracheal intubation could then be easily performed. It has been widely reported that a blind insertion of a SAD could be easily achieved in almost any patient position with very little need for airway manipulation [8]. Indeed, the first published reports of successful elective use of the classic laryngeal mask airway (LMA) in the prone position involved children undergoing radiotherapy [9, 10]. However, the reluctance of providers to utilize such an unconventional approach may in part be due to the following concerns [1]: the potential for difficult insertion [2]; inadequate ventilation from decreased lung compliance in the prone position [3]; the risk of regurgitation or aspiration because theoretically, prone positioning may increase intra-abdominal pressure [4]; the possibility of airway obstruction or laryngospasm, especially in children; and [5] dislodgement of the SAD [8, 11]. A review on the use of SAD in patients positioned other than supine, despite being supported mainly by published data on adult patients, reports that while airway events do occur, these episodes can be easily corrected or managed [8]. In addition, cumulative experience suggests that the use of SAD is feasible and useful in both elective and emergent airway management of patients in the prone position, but to prevent, recognize, and effectively solve critical airway events, safety precautions such as adequate training, careful patient selection, the use of best-suited devices, and the formulation of different rescue plans in case of failure, should be established [12–15]. With the advent of newer generation SADs (LMA® Supreme™, LMA® ProSeal™, LMA® Gastro™^,^ and Streamlined Liner of the Pharynx Airway™), improvements in their design allow for artificial ventilation in the prone position with fewer complications [14].

Other discussed methods of securing the airway in the prone position include direct laryngoscopy intubation and blind nasal intubation. These methods, however, may pose additional challenges to the provider, including difficult positioning for direct laryngoscopy and airway manipulation, multiple attempts, and the risk of traumatic nasal intubation that may lead to other problems like laryngospasm or aspiration [3]. In addition, complications may occur more frequently in children because of differences in their anatomy and physiology compared to adults.

The advantage of utilizing indirect laryngoscopy like a video laryngoscope (VL) over direct laryngoscopy is that the alignment of the oral, pharyngeal, and tracheal axes requires minimal movement of the head when using a VL.

We undertook a literature search and discovered a lack of available information on how anesthesiologists should manage such traumatic penetrating back injuries in children. The only indexed article we could find is that of Kim et al. [16]. They reported using a VL to successfully intubate a child with a stab wound to the back in a semilateral decubitus position. While there are several airway management options and techniques cited as being safe and effective when performed in the prone position, the available evidence is insufficient to widely recommend one method of securing the airway over another in emergency cases, and future high-quality multicenter studies are recommended [8, 12].

## 4. Conclusion

In conclusion, many factors must be considered in situations where children in the prone position need urgent airway management, especially in a resource-limited setting. We present this case report not only to share our experience that could be considered in emergencies like those described in our report but also to invite opinions from knowledgeable readers. We believe that the cornerstones of our successful outcome are fundamentally adequate planning, recognition of limitations, team coordination, and having a backup plan. More importantly, through our report, we would like to highlight that in our current age of innovative devices, modern medicine, and novel techniques, nothing can replace a sound knowledge and familiarity with established methods and the ability to improvise in a difficult clinical situation.

## Data Availability

The data used to support the findings of this study are included within the article.

## Abbreviations

CT: Computed tomography
IV: Intravenous
PVC: Polyvinyl chloride
ETT: Endotracheal tube
SAD: Supraglottic airway device
Mac: Macintosh
LMA: Laryngeal mask airway
VL: Video laryngoscope.
